# Supplementary material for: Olanzapine Modulate Lipid Metabolism and Adipose Tissue Accumulation via Hepatic Muscarinic M3 Receptor-Mediated Alk-Related Signaling
Source: Biomedicines. 2024 Jun 25;12(7):1403. doi: 10.3390/biomedicines12071403 (PMC11274235; doi:10.3390/biomedicines12071403)
Supplement: Supplementary file 1 [file biomedicines-12-01403-s001.zip › biomedicines-2973884-supplementary.pdf]

# Olanzapine Modulate Lipid Metabolism and Adipose Tissue Accumulation via Hepatic Muscarinic M3 Receptor-Mediated Alk-Related Signaling

**Yueqing Su [1,2], Chenyun Cao [3], Shiyan Chen [2,4], Jiamei Lian [2], Mei Han [2], Xuemei Liu [5] and Chao Deng [2,*]**

[1] Fujian Maternity and Child Health Hospital, College of Clinical Medicine for Obstetrics & Gynaecology and Paediatrics, Fujian Medical University, Fuzhou 350005, China

[2] School of Medical, Indigenous and Health Sciences, and Molecular Horizons, University of Wollongong, Wollongong, NSW 2522, Australia

[3] Department of Brain Science, Faculty of Medicine, Imperial College London, London SW7 2BX, UK

[4] Department of Neurology, The First Affiliated Hospital of Fujian Medical University, Fuzhou 350004, China

[5] School of Pharmaceutical Sciences, Southwest University, Chongqing 400715, China

* Correspondence: chao@uow.edu.au

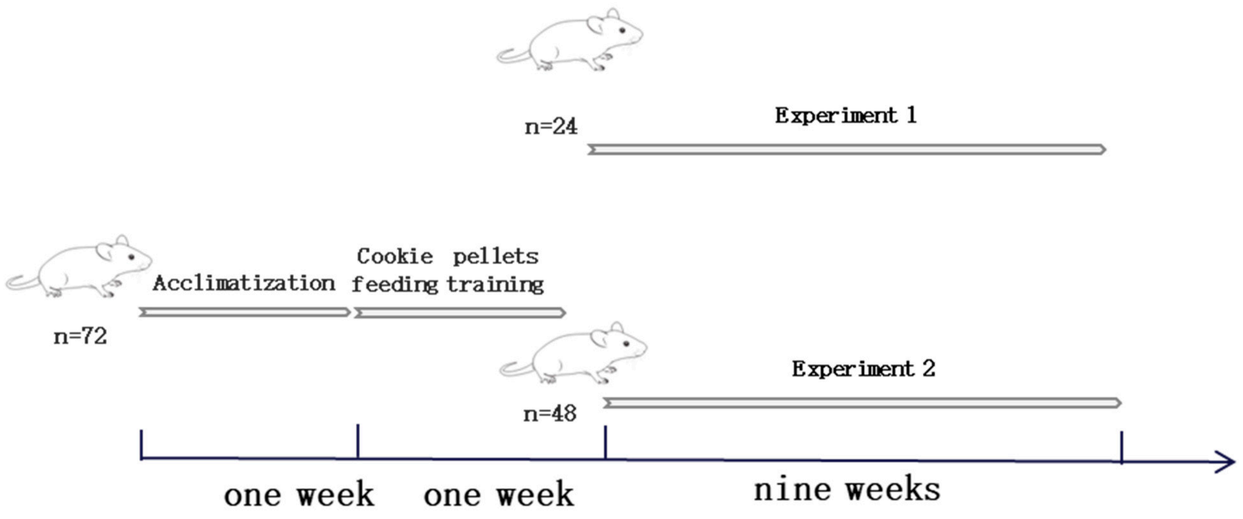


**Supplementary Figure S1.** Schedule of animal treatments. Following one week of acclimatization after arrival, rats were trained to take a cookie dough pellet (vehicle) for one week. Twenty four rats in Experiment 1 were randomly divided into two groups for olanzapine (6 mg/kg/day) or vehicle treatment (n=12/group, however one rat in the olanzapine group died during experiment), while forty eight animals in Experiment 2 were randomly divided into four groups for olanzapine (6 mg/kg/day), cevimeline (27 mg/kg/day), olanzapine (6 mg/kg/day) + cevimeline (27 mg/kg/day), or vehicle treatment (n=12/group), respectively.

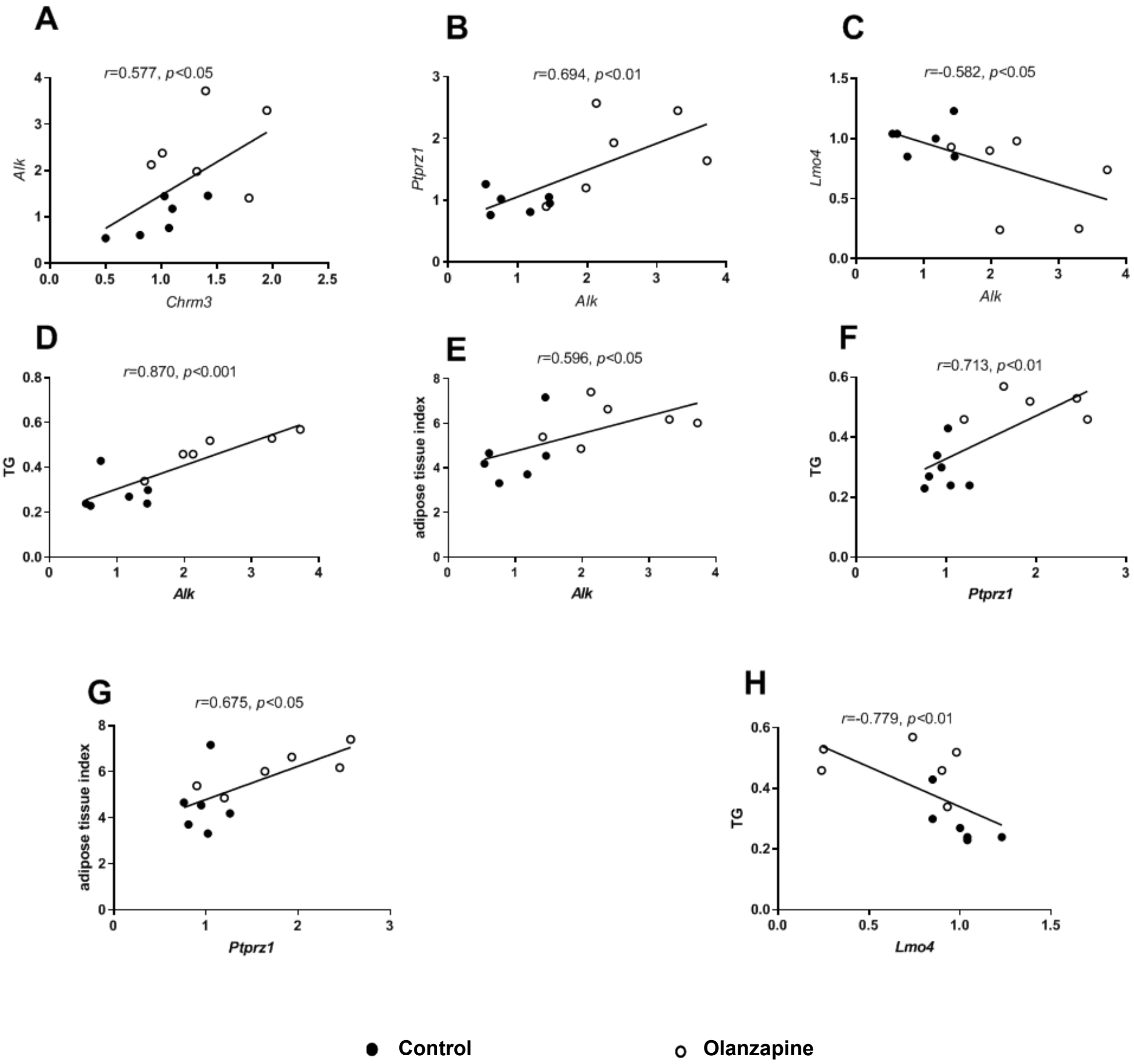


**Supplementary Figure S2.** Correlations between the expression of various genes and lipid characters after treatment with Olanzapine. Positive correlations between *Chrm3* and *Alk* (**A**), *Alk* and *Ptprz1* (**B**), *Alk* and TG (**D**), *Alk* and adipose tissue index (**E**), *Ptprz1* and TG (**F**), *Ptprz1* and adipose tissue index (**G**); Negative correlation between *Alk* and *Lmo4* (**C**), *Lom4* and TG (**H**).

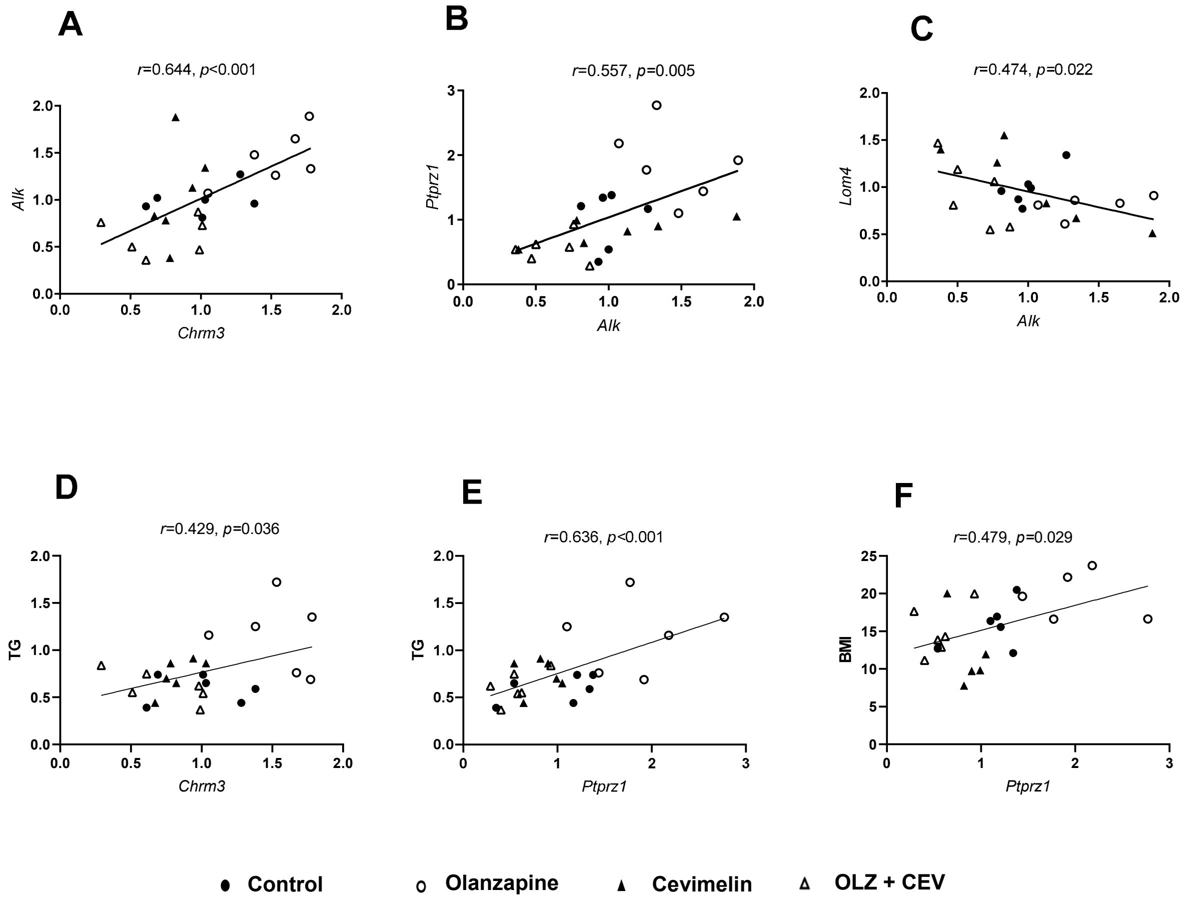


**Supplementary Figure S3.** Correlations between the expression of various genes and lipid characters after treatment with Olanzapine (a muscarinic M3 receptor antagonist) and/or Cevimeline. Positive correlations between *Chrm3* and *Alk* (**A**), *Alk* and *Ptprz1* (**B**), *Chrm3* and TG (**D**), *Ptprz1* and TG (**E**), *Ptprz1* and BMI, (**F**); A negative correlation between *Alk* and *Lmo4* (**C**).
